# The Physical Activity and Cancer Control (PACC) framework: update on the evidence, guidelines, and future research priorities

**DOI:** 10.1038/s41416-024-02748-x

**Published:** 2024-06-27

**Authors:** Lin Yang, Kerry S. Courneya, Christine M. Friedenreich

**Affiliations:** 1 Department of Cancer Epidemiology and Prevention Research, Cancer Care Alberta, Alberta Health Services, Calgary, AB Canada; 2 Departments of Oncology and Community Health Sciences, Cumming School of Medicine, University of Calgary, Calgary, AB Canada; 3 Faculty of Kinesiology, Sport and Recreation, College of Health Sciences, University of Alberta, Edmonton, AB Canada

**Keywords:** Cancer epidemiology, Cancer prevention

## Abstract

**Background:**

We proposed the Physical Activity and Cancer Control (PACC) framework in 2007 to help organise, focus, and stimulate research on physical activity in eight cancer control categories: prevention, detection, treatment preparation/coping, treatment coping/effectiveness, recovery/rehabilitation, disease prevention/health promotion, palliation, and survival.

**Methods:**

This perspective paper provides a high-level overview of the scientific advances in physical activity research across cancer control categories, summarises current guidelines, updates the PACC framework, identifies remaining and emerging knowledge gaps, and provides future research directions.

**Results:**

Many scientific advances have been made that are reflected in updated physical activity guidelines for six of the cancer control categories apart from detection and palliation. Nevertheless, the minimal and optimal type, dose, and timing of physical activity across cancer control categories remain unknown, especially for the understudied population subgroups defined by cancer type, age, race/ethnicity, and resource level of regions/countries.

**Conclusion:**

To achieve the full benefit of physical activity in cancer control, future research should use innovative study designs that include diverse at-risk populations and understudied cancer sites. Additionally, effective behaviour change strategies are needed to increase physical activity levels across populations that use implementation science to accelerate the translation from evidence generation into practical, real-world interventions.

## Introduction

**Fig. 1:**
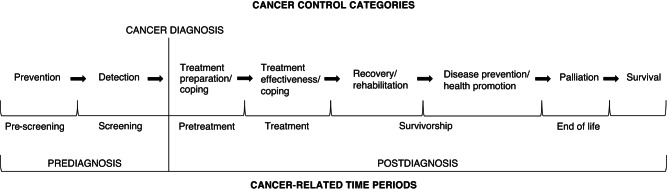
Physical Activity and Cancer Control (PACC) framework (reproduced from [3]^a^). ^a^This article was published in *Seminars Oncology Nursing* journal, Copyright Elsevier (2007).

The cancer care continuum is a multi-phase, sometimes cyclic process that may include multiple treatment modalities, cancer progression, recurrence and new primary cancers and that necessitates a range of cancer control categories depending on various factors including the patient and setting [1]. While the major focus in cancer treatment and care has been on clinical interventions, only in the past few decades has the role of lifestyle factors, including dietary intake and physical activity, been considered in cancer control. The first research studies that examined how physical activity could be involved in cancer etiology were published in early 1980s with limited research until the 1990s. Given that this field was relatively new and unstructured, we were motivated to publish an organizational framework for research on physical activity across the cancer experience (PEACE) to help provide structure to this emerging research area [2]. In 2007, we updated that framework with the Physical Activity and Cancer Control (PACC) framework [3] to organize, focus, and stimulate further research on physical activity and cancer control (Fig. 1). Eight cancer control categories (eight populations at risk) on the cancer care continuum were specified in the PACC framework in six cancer-related time periods including two pre-diagnosis (prevention, detection) and four post-diagnosis (pre-treatment, treatment, survivorship and end of life). This framework has been seminal in structuring and focusing research on physical activity in cancer control to specific time points.

The purpose of this review paper was to assess the current state of scientific evidence on physical activity across the entire cancer continuum from prevention to survival to identify: 1) existing knowledge gaps; 2) opportunities for future research and 3) needs for further physical activity/exercise guideline development. We conducted a narrative review to assess the updated current evidence of the role of physical activity in cancer control in accordance with the PACC framework (Supplementary Materials). We searched for published systematic reviews in physical activity/exercise and cancer control in the past five years in MEDLINE by March 19, 2023, and updated the search on April 27, 2024. When systematic reviews were not available for a component of the PACC framework, we included the most current literature based on our literature search to identify and assess the emerging evidence and to identify future research directions.

Throughout this review paper, we use the term “physical activity” which includes all forms of movement done in occupational, recreational, household and transportation settings. For post-diagnosis components of the PACC framework (e.g., before, during and after treatment) where research studies frequently investigated exercise interventions, the focus of the evidence review is mainly on “exercise” which is a subset of physical activity that is planned, structured, and done repeatedly to improve or maintain physical fitness. Herein we describe the current state of evidence on physical activity by cancer control category followed by a summary of the current physical activity guidelines. We then identify the gaps in knowledge and identify priority areas for future research.

## Evidence review

### Physical activity and cancer prevention

The body of evidence on the etiologic role of physical activity in cancer prevention has markedly increased since 2007, when colon, breast, endometrial, prostate and lung were the main cancer sites that had been investigated [3]. The evidence on risk reduction for cancer of the gallbladder, liver, kidney, small intestine, myeloid leukemia, myeloma, and non-Hodgkin lymphoma is now accumulating [4]. Highest vs. lowest leisure-time physical activity was associated with a relative cancer risk reduction ranging between 8 to 25%, with the level of evidence deemed strong for bladder, breast, colon, endometrium, esophagus, gastric and renal, moderate for lung, and limited for hematologic, head and neck, ovary, pancreas, prostate, brain, thyroid and rectal cancer [5]. Recent review articles have also begun to synthesize evidence for understudied cancer types and different dimensions of physical activity, reporting inconsistent associations between physical activity and risk of testicular cancer [6], lower risk in colon (26%) but higher risk (115%) in lung cancer associated with occupational physical activity [7, 8], lower risk in endometrial, colon and breast cancers associated with transport-related physical activity (1–9%) [9] and lifetime physical activity (18–25%) [10], lower risk in colon and breast cancers associated with physical activity at young age (19–33%) [10]; and have examined potential risk stratifications by menopausal status [11] and by family history for breast [12] and colorectal cancer [13], and potential biological mechanisms involved for breast cancer [14, 15].

It is worth noting that nearly all research evidence on physical activity for cancer prevention is from observational epidemiologic studies given the cost, feasibility and time requirements that would be required for randomized controlled trials. To the best of our knowledge, no randomized controlled exercise trials reported cancer incidence as the primary endpoint. Consequently, the focus has been on exercise intervention trials that have examined hypothesized biological pathways [16, 17] that could explain how physical activity reduces the risk of developing cancer. Observational studies have suggested the relationship between physical activity and specific cancer risk differs by the magnitude of the relative risk reduction as well as the shape of the dose-response [18]. Leisure-time physical activity within the recommended level (7.5–15 MET hours/week) was associated with 6–29% risk reduction among several cancers, including colon, breast, endometrial, kidney, myeloma, liver and non-Hodgkin lymphoma (in women). Beyond the recommended level, physical activity continued to further reduce risk in some but not all cancers, likely suggesting fundamental differences in the underlying biological mechanisms for different cancer types [18]. However, the number of trials testing the minimal and optimal dose (type, frequency, intensity, and duration) of exercise for cancer prevention remains small [19–23]. Additionally, these trials have focused primarily on aerobic exercise with only a limited emphasis on resistance (i.e., muscle strengthening activity) as well.

Muscle-strengthening activities are not well captured by self-reported questionnaire or accelerometer-based devices that are commonly used in large cohort studies. A small body of evidence from observational studies found muscle-strengthening activity was associated with inconsistent findings for the risk of colon and bladder cancer [24–27], reduced risks for kidney cancer [24–26], and limited evidence regarding the risks for cancers of the lung, pancreas, prostate and rectal [26]. Muscle-strengthening activities involving major muscle groups could influence metabolic function [28], inflammation [29], and sex hormones [30–32], which are biological mechanisms associated with developing cancer [33]. Currently, it is unknown whether muscle-strengthening activity alone, or combined with aerobic activity, contributes to cancer risk reduction.

Sedentary behavior, independent of physical activity, has been associated with an increased risk of colon, endometrial and lung cancer [34]. Sedentary behavior is hypothesized to operate through the metabolic function pathway to influence cancer risk [33]. Given the methodologic limitations in measuring the bouts and duration of sedentary behavior at the population level and the lack of available intervention tools to interrupt or reduce sedentary behavior, generating observational or interventional data to examine and elucidate the potential relationship between sedentary behavior and cancer risk remains limited.

### Physical activity and cancer detection

Limited research has examined the role of physical activity on cancer detection. Physical activity has at least two ways to influence cancer detection: 1) indirectly by influencing cancer screening behavior (i.e., higher uptake of cancer screening which could result in an early stage at diagnosis), and 2) directly by influencing the sensitivity and/or specificity of cancer screening tests [3]. Several studies indicated that physical activity, among other lifestyle behaviors, was associated with higher uptake of colorectal screening [35, 36]. Observational studies that examined cancer stage at diagnosis found physical activity was associated with higher odds of late-stage lung cancer diagnosis [37] and no association with stage at breast cancer diagnosis [38]. With respect to cancer screening tests, observational and intervention studies have found no association between physical activity and mammographic density [39–42]. Meanwhile, physical activity, especially acute exercise may increase serum prostate-specific antigen (PSA) concentration [43, 44] leading to false PSA levels, but habitual physical activity may be associated with lower PSA concentration [45]. More recently, a case study reported that the supervision of exercise may be a cancer detection test for metastatic disease by uncovering symptoms that suggest recurrence or metastatic spread such as pain, neurological issues, or functional issues [46]. These “exercise-detected” symptoms may allow for earlier detection of recurrence or metastatic spread.

### Physical activity and treatment preparation/coping

Using physical activity as preparation to increase readiness prior to cancer treatment, or cancer prehabilitation, has gained significant clinical interest [47]. The concept of “*prehabilitationc*” was first scientifically documented in 1946, in a non-clinical setting, as a total body exercise training program that the British Army developed to increase the physical health of young recruits, i.e., to qualify for conscription [48]. In the oncology setting, physical activity for cancer prehabilitation has the potential to: 1) improve physical health to endure or become eligible for cancer treatment, 2) improve psychological health to cope with the diagnosis and impending cancer treatment, 3) prevent treatment-induced toxicities and improve treatment completion and recovery, and 4) influence the tumor biology and microenvironment directly to improve treatment efficacy or delay the need for treatment. Over 30 review articles have been published on the topic of physical activity for cancer prehabilitation. Existing systematic reviews were mostly focused on: selective cancer sites (majority in lung cancer surgery) [49–60], cancer site-specific treatment side-effects [61–63], or selective exercise intervention modalities (HIIT or combined aerobic and resistance training) [64–67]. Importantly, many prehabilitation studies included cancer patients undergoing neoadjuvant therapy [49, 51–53, 67–72] or undergoing other systematic therapies (chemotherapy and radiation therapy) [73, 74], which overlaps with the cancer control category of physical activity and treatment effectiveness/coping. While the combination of patients before and during treatment does not allow for investigation into physical activity and cancer control category-specific outcomes, both cancer control categories are situated in the clinical setting, i.e., likely using similar or overlapping intervention delivery strategies/pathways.

Given the relatively small volume of primary research in treatment preparation/coping, it is not surprising that findings from previous systematic reviews were inconclusive. Our recent scoping review reports that the number of primary studies on physical activity for cancer prehabilitation consists of 32 interventions and 12 observational studies using heterogeneous intervention designs and physical activity assessments [75]. All previous studies on physical activity and cancer prehabilitation enrolled patients waiting for planned cancer treatment, therefore it is unknown whether physical activity may influence treatment decision especially in settings where multiple treatment options exist. Prehabilitation exercise appears to improve health-related fitness but the evidence to improve clinical outcomes is lacking [75]. Notably, nearly all studies conducted in the oncology setting to investigate the role of physical activity for prehabilitation have focused on surgical treatment, presenting a major knowledge gap and opportunities to increase evidence generation and translation to realize the full potential benefits of physical activity to prepare cancer patients facing non-surgical treatment modalities.

### Physical activity and treatment effectiveness/coping

Many randomized controlled trials have been conducted in this cancer control category testing exercise dose in accordance with the generic physical activity guidelines. For example, for cancer patients undergoing curative treatment, there is evidence from over 70 systematic reviews of exercise interventions. Notably, the endpoints of these interventions were largely patient-oriented instead of oncologic outcomes, i.e., improved fatigue, cardiorespiratory fitness, muscle strength, physical function, body composition, quality of life, depression and anxiety, sleep, and cognitive function. Physical activity may influence treatment effectiveness/coping through: 1) a direct effect on tumor growth and metastasis, 2) improved treatment completion rate, 3) improved treatment efficacy, 4) managing treatment-induced toxicities, and 5) improved physical, psychosocial outcomes and quality of life. However, most of these hypothesized effects have not been fully investigated.

In this cancer control category, exercise interventions remain exploring feasibility measures in different treatment settings [76, 77], understanding the potential to prevent cardiotoxicity [78–80], and accumulating data on physical, psychosocial outcomes and quality of life [81, 82]. Limited data are available on the impact of exercise intervention on “treatment effectiveness”, such as chemotherapy completion or dose intensity, cancer treatment response, or cancer progression, recurrence and survival [83]. Furthermore, there is significant heterogeneity across exercise intervention studies in their study designs, interventions, and study populations making it difficult to recommend the minimal and optimal type and dose of physical activity during cancer treatment. Nevertheless, interest is growing to investigate exercise as a cancer treatment; considerations related to observational and experimental study designs for specific clinical oncology settings have been detailed elsewhere [84, 85].

Another important consideration in, but not limited to, this cancer control category is the potential harm of exercise. A recent systematic review and meta-analysis included 129 published and unpublished controlled trials comparing exercise interventions versus usual care in adults with cancer scheduled to undergo systemic treatment to synthesize evidence on adverse events, health-care utilization, and treatment tolerability and response [86]. This meta-analysis reported higher risks of serious adverse events, thromboses, and fracture, lower risk of fever, and higher relative dose intensity of systemic treatment in intervention versus control. Their findings indicated uncertainty regarding the harms of exercise in cancer patients undergoing systemic treatment, and concluded that insufficient data existed on the harms to make evidence-based risk-benefit assessments of the application of structured exercise in this population.

### Physical activity and cancer survivorship (recovery/rehabilitation and disease prevention/health promotion)

**Fig. 2:**
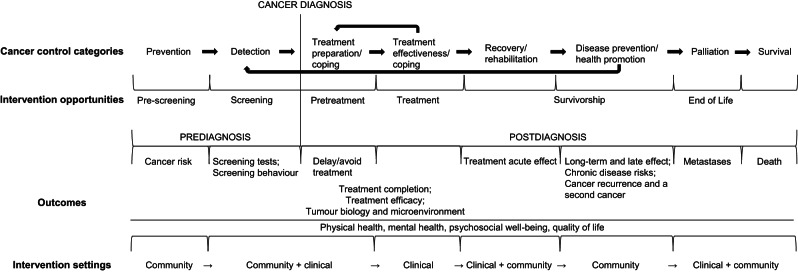
Updated physical activity and cancer control framework.

Many systematic reviews have been published on the topic of physical activity and cancer survivorship which includes the two cancer control categories of “recovery/rehabilitation” and “disease prevention/health promotion”. Separating research conducted in these two cancer control categories is difficult because of the lack of clear definitions for patient characteristics and outcome assessment. “Recover” defined by the US National Cancer Institute is “to become well and healthy again” [87], and “rehabilitation” is defined as “a process to restore mental and/or physical abilities lost due to injury or disease, in order to function in a normal or near-normal way” [88]. Hence, the recovery/rehabilitation category is the acute phase (within six months) after treatment completion [3] with the goal for cancer patients to regain function and reach normalcy. Therefore, the goals of physical activity after cancer treatment completion for recovery/rehabilitation are to: 1) reduce acute treatment toxicities, 2) improve physical function, 3) improve psychosocial outcomes, to 4) improve quality of life and regain normalcy. With respect to “health promotion”, defined by WHO as “the process of enabling people to increase control over, and to improve, their health”, and “disease prevention”, the focus is on specific efforts aimed at reducing the development and severity of chronic disease and other morbidities [89, 90]. Therefore, the goals of physical activity after cancer treatment completion for disease prevention/health promotion are to: 1) manage long-term and late effects of cancer and cancer treatment, 2) improve psychosocial outcomes and quality of life, 3) reduce the risks of chronic diseases, and 4) reduce the risk of cancer recurrence, and a second primary cancer. Accordingly, we updated the PACC framework to include specific outcomes and intervention settings for each cancer control category (Fig. 2).

Over 1000 exercise trials have been conducted to examine the effect of exercise on outcomes during the post-treatment survivorship period. Nevertheless, common cancers such as breast [91, 92], prostate [93–95], colorectal [96, 97], and lung cancers [98, 99] are heavily investigated, some studies on childhood cancers [100, 101], while there is a paucity of data on less common cancers [102] including survivors of adolescent and young adult cancer [103]. Admittedly, the potential feasibility of this type of trial is low in less common cancers, especially when randomized controlled trials tend to be designed and powered to generate evidence on a single primary outcome [104, 105]. Even for cancer types/sites that are more frequently investigated, few data are available on the effect of physical activity on chronic disease risks among cancer survivors. A few reviews summarized the positive effect of exercise on the cardiovascular system [106], C-reactive protein as an inflammatory biomarker [107], blood lipid profile [108], fasting insulin levels [109], and metabolic function [110] among breast cancer survivors as biologic pathways involved in physical activity and breast cancer outcomes. These biologic pathways also operate on the development of chronic disease among cancer survivors. Cancer treatment can exacerbate the existing comorbidities and increase the risk of new comorbidities in cancer survivors [111, 112], many of which can be potentially prevented and managed by physical activity. Currently, the natural history of comorbidities in relation to physical activity is largely unknown in cancer survivors, representing a clear gap of future studies to inform the timing, type, frequency, intensity, and duration of physical activity that should be considered in exercise interventions aiming at disease prevention.

Although most identified systematic reviews remain focused on synthesizing the effect of exercise on acute, long-term, and late effect of cancer, several reviews have begun to explore how to design physical activity behavior change interventions among cancer survivors [113–123] including digital approach [124]. These intervention designs include both clinical [125, 126] and community [127–131] settings, but have mostly used individual-based approaches. Typical strategies of health promotion and disease prevention are population-based interventions that aim to address social determinants and health inequity simultaneously [90]. However, how to design population-based interventions with evidence-based risk stratification strategies that target cancer-specific outcomes remains unknown.

### Physical activity and palliation

Palliative care aims to improve quality of life and help reduce pain in people who have a serious or life-threatening disease [132]. The body of literature on physical activity in the cancer palliation setting is increasing yet remains limited. The largest most recently published systematic review identified 22 exercise interventions explicitly in the cancer palliative care setting that improved quality of life, health-related fitness and fatigue [133–135]. Other reviews reported similar benefits of exercise interventions among patients with advanced cancer [136–145] or with cancer metastasis [146–149] on physical and psychological health and quality of life outcomes. The 2007 PACC framework proposed that in the palliative care setting, physical activity may help cancer survivors manage symptoms, improve mobility, slow functional decline, and maintain quality of life at the end of life [3]. Importantly, the survival of certain advanced cancers has improved to the point where a diagnosis of advanced cancer is no longer an acute “end of life” phase [150]. In addition, early palliative care may even prolong cancer survival [151]. While exercise appears feasible in this setting, more evidence is needed to understand the potential benefits and harms of physical activity in patients diagnosed with advanced cancer, especially those with bone metastases [152]. More importantly, given the potential of palliative care to prolong cancer survival, further research in physical activity and cancer palliation should consider clinical outcomes such as disease progression and metastasis [84]. Given the severe symptoms associated with advanced cancer and its treatment, such as fatigue, pain and muscle wasting, exercise trials in this population are more challenging and should consider the physical and psychological capability of cancer survivors to avoid exacerbating any symptoms and mental distress.

### Physical activity and survival

There is limited to moderate evidence for physical activity to reduce cancer-specific and all-cause mortality for breast, colorectal and prostate cancer [5]. Notably, the mortality risk reduction associated with physical activity is estimated to range between 30% for cancer-specific mortality and 32% reduced risk of all-cause mortality [153]. A few ongoing randomized controlled trials are examining the effect of exercise intervention on survival outcomes among colorectal [154] and prostate [155] cancer survivors. Given the challenges in cost, recruitment and time requirements in such trials, the minimal and optimal type and dose of exercise to improve cancer survival remains unknown. One large scale, on-going observational study in breast cancer is assessing physical activity, sedentary behavior and health-related fitness using both direct measurements as well as self-report to examine the association with survival outcomes with greater precision [156, 157]. In addition, the effect of exercise on inflammatory, insulin, and metabolic pathways involved in physical activity and cancer survival is hypothesized [110], however, current evidence is limited to select high risk populations, or to monitor the biologic response to exercise among cancer survivors. This topic area is being assessed in some of the on-going trials and observational studies [154–156, 158].

## Physical activity guidelines

As the evidence base on physical activity in cancer control has accumulated, national and international agencies have developed and updated their physical activity guidelines. With more targeted research, it has also been possible to begin developing more specific guidelines for the cancer control categories with some developed for all but cancer detection and palliation. We have included examples of current guidelines that provided recommendations on quantified physical activity/exercise and cancer in each of the cancer control categories (Table 1).

**Table 1:** Examples of international guidelines/recommendations/position statements on physical activity and exercise across the cancer control categories.

Cancer control category	Guidelines/recommendations/position statement
Prevention	ACS, HHS, WCRF: 150–300 min of moderate-intensity aerobic activity, 75–150 min of vigorous aerobic activity, or an equivalent combination of each intensity each week.
Detection	None
Treatment preparation/coping	ASCO: may recommend preoperative exercise for patients undergoing surgery for lung cancer.
Treatment coping/effectiveness	ACS, ACSM, ASCO: 150–300 min/week of moderate-intensity activity or 75–150 min/week vigorous-intensity activity or a combination of the two intensities, and muscle-strengthening two or more days per week to improve quality of life during treatment.
Recovery/rehabilitation	ACSM: 150 min/week aerobic exercise and twice weekly strength training to manage the acute, long-term and late effect of cancer.
Disease prevention/health promotion	ACSM: 150 min/week aerobic exercise and twice weekly strength training to manage the acute, long-term and late effect of cancer.
Palliation	None
Survival	ACS, ACSM: 150–300 min/week of moderate-intensity activity or 75–150 min/week vigorous-intensity activity or a combination of the two intensities, and muscle-strengthening two or more days per week to improve survival for during long-term disease-free living or stable disease.

*ACS* American Society of Cancer, *ASCO* American Society of Clinical Oncology, *ACSM* The American College of Sports Medicine, *HHS* U.S. Department of Health and Human Services, *WCRF* World Cancer Research Fund International.

### Cancer prevention

For physical activity and cancer prevention, the World Cancer Research Fund (WCRF) [159] in 2018 recommended that adults be active daily and do at last 150 min of moderate physical activity or at least 75 min of vigorous physical a week which aligned with the 2008 Physical Activity Guidelines for Americans [160] and the 2010 World Health Organization’s (WHO) guidelines on physical activity [161]. The 2018 Physical Activity Guidelines for Americans updated their recommendation to 150 to 300 min of moderate-intensity aerobic activity, 75–150 min of vigorous aerobic activity, or an equivalent combination of each intensity each week for cancer prevention, which were adopted by the 2020 American Cancer Society (ACS) guidelines for reducing the risk of developing cancer [162] and the 2020 World Health Organization’s (WHO) recommendation [163]. These updated guidelines have recognized that more physical activity (i.e., up to 300 min/week) confers additional benefits whilst emphasizing that benefits are also attained at 150–300 min of weekly activity. Generic physical activity guidelines, e.g., those of the WHO [163], or USA [164], recommend twice-weekly muscle-strengthening activity in addition to aerobic activity for health promotion and the prevention of other chronic disease such as cardiovascular disease and Type 2 diabetes [163]. Muscle-strengthening activity, however, is currently not included in cancer prevention guidelines because of the lack of evidence. Another physical activity related recommendation in generic physical activity guidelines [163] is to limit sedentary behavior, which is not currently included in guidelines specific to cancer prevention.

### Cancer treatment

The American College of Sports Medicine (ACSM) published updated exercise guidelines for cancer survivors during and after treatment in 2019 [102]. In 2022, the American Society of Clinical Oncology (ASCO) developed guidelines stating that oncology providers should recommend regular aerobic and resistance (muscle-strengthening) exercise for patients during active treatment with curative intent and preoperative exercise for patients undergoing surgery for lung cancer [165]. Similar guidelines have been developed for activity during and after treatment in several other countries worldwide (e.g. Australia, UK) [166, 167].

The 2019 ACSM updated exercise guidelines for cancer survivors stated that exercise training was generally safe and well tolerated during and after cancer treatment, and recommended 150 min/week aerobic exercise and twice weekly strength training to manage the acute, long-term and late effects of cancer [102]. More specific guidelines were provided for particular symptoms and side effects including fatigue, anxiety, depression, and sleep quality [102].

The 2022 American Cancer Society (ACS) nutrition and physical activity guidelines [168] for cancer survivors agreed with the WHO 2020 guidelines for populations with chronic disease. Both organizations currently recommend 150–300 min/week of moderate-intensity activity or 75–150 min/week vigorous-intensity activity or a combination of the two intensities, and muscle-strengthening two or more days per week. These recommendations are aimed at improving quality of life during cancer treatment and long-term survivorship for survivors who are disease-free or have stable disease [168].

### Cancer survivorship

The ACSM 2019 guidelines were developed based on evidence generated from randomized controlled trials that examined the effect of exercise on a list of cancer-related health outcomes with a high degree of clinical relevance [102]. It is important to note that these outcomes of acute, long-term and late effects of cancer have variable trajectories [169].

Consequently, the randomized controlled trials in the evidence syntheses included cancer survivors at both recovery/rehabilitation and disease prevention/health promotion categories.

For cancer survivors who are disease-free or living with stable disease, the 2022 ACS guidelines recommend physical activity to reduce cancer-specific and all-cause mortality for specific cancer types (breast, upper aerodigestive and digestive system, genitourinary, gynecologic, lung, hematological, and childhood cancer survivors) [168]. Besides evidence on survival, the ACS guidelines attempted to synthesize evidence on physical activity and the risk of cancer recurrence and a second cancer [168]. Currently, there is preliminary evidence synthesized from two randomized controlled trials on physical activity to reduce the risk of breast cancer recurrence [170], but in general there is no data on other cancer sites/types or the risk of a second primary cancer.

## Future research

This review of the current state of evidence on the role of physical activity in cancer control has revealed that evidence has accumulated unevenly across the cancer continuum with most evidence on cancer prevention, during cancer treatment, and rehabilitation; moderate research on cancer survival; and only limited evidence in cancer detection and palliation. Furthermore, across all cancer control categories in the PACC framework, it remains unknown which populations may benefit the most from physical activity to improve cancer-related outcomes and the minimal and optimal type, frequency, intensity, and duration of physical activity to achieve such benefits (Table 2). Substantial biological and behavioral heterogeneities exist within populations in terms of how they respond to physical activity. Some initial research has been conducted demonstrating the potential effect of physical activity on colorectal cancer risk reduction irrespective of cancer genetic predisposition [171]. Much more observational and intervention epidemiologic research is required to understand the biologic mechanisms whereby physical activity may reduce cancer risk and to identify biologic markers that could be used to identify, monitor, and evaluate individuals who could benefit from physical activity. Thus far, most of the existing evidence and guidelines have been derived from and pertain to high-income countries. While the level of physical activity remains higher in low- and middle-income countries than in high income countries [172], there is a global trend of declining physical activity that was exacerbated by the COVID-19 pandemic [173]. Future research is needed in low- and middle-income countries to refine the evidence of cancer prevention and control in relation to physical activity and to reverse this declining trend. In addition, programs and policies that are targeted, relevant, and feasible in these low- and middle-income countries are needed that will encourage physical activity and reverse the declining levels of activity that accompany industrialization.

**Table 2:** Cancer outcomes and future directions in physical activity and cancer control.

	Outcomes with evidence	Future directions
Prevention	Cancer risk, cancer stage, cancer subtype.	Role of muscle strengthening activity in cancer risk reduction Biological mechanisms involved in physical activity and cancer risk Evidence on physical activity and risk reduction for rare cancers
Detection		Effect of physical activity on cancer screening behavior Effect of physical activity on sensitivity and/or specificity of cancer screening tests
Treatment preparation/coping	Health-related fitness in some cancers, postoperative length of hospital stays and complication in lung cancer.	Effect of physical activity on postsurgical complications Effect of physical activity on length of hospital stay Evidence on physical activity during treatment preparation/coping in non-surgical settings Effect of physical activity on treatment-induced toxicities prevention Effect of physical activity on tumor biology and microenvironment Effect of physical activity on treatment completion and efficacy Effect of physical activity on treatment delay and/or avoidance
Treatment coping/effectiveness	Health-related fitness, cancer symptom management, cancer and treatment related side effects.	Effect of physical activity on treatment-induced toxicities management Effect of physical activity on tumor biology and microenvironment Effect of physical activity on treatment completion and efficacy
Recovery/rehabilitation	Health-related fitness, cancer symptom management, cancer and treatment related side effects.	Evidence on physical activity during recovery/rehabilitation in less common cancers
Disease prevention/health promotion	Health-related fitness, cancer symptom management, cancer and treatment related late effects.	Effect of physical activity on cancer recurrence and a second primary cancer Effect of physical activity on the risk of other chronic diseases Evidence on physical activity during disease prevention/health promotion in less common cancers
Palliation	Health-related fitness, cancer symptom management, cancer and treatment related late effects.	Effect of physical activity on the risk of disease progression and cancer metastases
Survival	All-cause and cancer specific survival in breast, prostate and colorectal cancers.	Effect of physical activity on other disease-specific survival Biological mechanisms involved in physical activity and survival outcomes Evidence on physical activity and survival in other cancers
All cancer control categories	Evidence generation • What are the optimal types, frequencies, intensities, and durations of physical activity for improved outcomes in cancer prevention and control? • What is the role of sedentary behavior in cancer prevention and control? • How can we improve the precision of intervention content (risk-stratification to identify population subgroups with the greatest gain from physical activity intervention)? • How can we improve the precision of intervention delivery (implementation model personalized to individuals interacting with the complex social system)? • How do we design and test interventions in populations to increase their physical activity when we have limited evidence accumulated because these populations have individual, physical, environmental, and financial barriers to increasing physical activity?	
	Evidence translation • How should we use a whole-systems approach to develop population behavior change interventions? • How do we identify and engage key stakeholders by setting (clinical and/or community)? • Is there a logic model for intervention delivery to inform process measures that we could incorporate in trial implementation, monitoring, and evaluation?	

Health-related fitness: cardiorespiratory fitness, muscular strength and endurance, body composition, flexibility.

Cancer symptoms, side effects and late effects of cancer: including but not limited to anxiety, bone health, cardiotoxicity, chemotherapy-induced peripheral neuropathy, cognitive function, depression symptoms, falls, fatigue, health-related quality of life, lymphedema, nausea, pain, physical function, sexual function, sleep, treatment tolerance.

### Observational research

Observational studies are the cornerstone to generate evidence that can inform exercise dose in interventional studies [174, 175]. It is important to understand the minimal and optimal dose of exercise that can benefit targeted outcomes for individuals diagnosed with cancer, who face multi-faceted demands and time constraints. Many exercise interventions for cancer survivors follow the generic physical activity guidelines. There is a paucity of data generated from observational studies that is specific to cancer survivors. The effect of physical activity on the range of cancer, health-related, and healthcare utilization outcomes, is particularly unexplored in the three most clinically relevant cancer control categories: treatment preparation/coping, treatment coping/effectiveness, and palliation. Thus, generating this evidence is critical to inform the optimal design of clinical exercise programs.

### Intervention research

Clinical exercise trials that use experimental designs can serve a range of purposes to test the effect of physical activity on a specific outcome, to investigate the underlying biological mechanisms, to determine the optimal dose of exercise intervention, or to compare different exercise modalities to address causality in physical activity and cancer control. Given the accumulating evidence supporting the benefits of exercise on patient-centered outcomes and the current knowledge gaps, more intervention studies after diagnosis are needed in under-studied patient populations that are adequately powered to examine clinically important outcomes (such as treatment efficacy, recurrence, and survival) and to explore the heterogeneity in patient behavioral and biological responses to exercise. This research is needed before more definitive and targeted guidelines can be developed for health care providers to prescribe exercise to cancer patients and survivors.

Sustaining physical activity is challenging, which makes the investigation into long-term physical activity interventions and cancer outcomes extremely challenging. Effective intervention design should consider two domains of precision: 1) the intervention content (i.e., type and dose of physical activity specific to cancer-related outcomes) and the intervention delivery (i.e., individuals interacting with the complex social system). Hence, behavior change research is required to understand the behavioral mechanisms involved in exercise intervention delivery for cancer survivors. Behavior change techniques targeting individual and structural barriers to increase physical activity, ideally, should be fully integrated into clinical exercise trials in the oncology setting to enhance the intervention fidelity.

Future intervention studies should also consider adequate adverse event reporting for risk-benefit assessments, particularly among patients with high symptom burdens. Currently, adverse event tracking tools exist in the oncology setting, such as the European Organisation for Research and Treatment of Cancer (EORTC) Item Library for patient-reported outcomes and the Common Terminology Criteria for Adverse Events (CTCAE) for clinician reporting [176]; however their application in exercise trials is yet to be standardized.

Meanwhile, modern societies continue to become increasingly sedentary [177]. One important factor contributing to this sedentary trend was the sole focus on increasing structured, moderate-to-vigorous intensity physical activity in previous interventions [178]. Such interventions were indeed useful for socially advantaged populations but likely widened the equity gap between high resource populations and those facing barriers related to social and environmental constraints created by limited time, affordability, access to facilities, and low neighborhood walkability [179]. Novel approaches such as home-based interventions addressing the social and environment determinants of physical activity are needed to narrow the equity gap between high and low resource populations. From a behavior change perspective, interventions to increase physical activity could target sedentary behavior to interrupt prolonged sitting to increase physical activity. Developing tools and methods to assess domain-specific physical activity (including both aerobic and muscle-strengthening activities) and sedentary behavior at the population level, as well as intervention tools to interrupt and reduce sedentary behavior, are necessary to begin generating efficacy evidence on the cancer prevention potential of targeting sedentary behavior.

### Biologic mechanisms research

Whilst some observational [180] and experimental research [19–23] has examined the underlying biologic mechanisms that are operative between physical activity and cancer control outcomes, there remains a dearth of evidence regarding these putative mechanisms. A key recommendation for future research is to embed assessments of biologic mechanisms into future observational and exercise intervention trials.

### Translational research

Primary prevention is arguably the most challenging timepoint in the cancer care continuum for translating from evidence to implementation because of the inherent difficulties in evaluating effective interventions aimed at reducing cancer incidence in the population [181]. Cancer prevention requires a sufficient evidence base, political will to fund programs to address the prevention potential, and a social strategy or plan by which we apply our knowledge to initiate or improve programs [182]. Current efficacy evidence on the role of physical activity in cancer etiology, although not yet fully elucidated, is sufficient to inform policy changes and to develop strategies for population health interventions to increase physical activity for primary cancer prevention. Nevertheless, evidence is lacking on how to best design interventions to increase physical activity at the population level [183]. Whilst programs for physical activity exist, chronic conditions and low resources pose physical, financial and environmental barriers to participation [184]. These barriers are remarkably common among individuals at disadvantaged socioeconomic positions who are also at higher risks of cancer and, therefore, the highest needs for behavior change. Hence, future research should consider evaluating physical activity as the endpoint in effectiveness trials that take into consideration equity parameters to fully understand the context of behavior change.

Several guidelines exist on physical activity during cancer treatment and cancer survivorship. While there remains a dearth of evidence for specific components across the cancer continuum, sufficient evidence has been accumulated for several time points across this cancer continuum. More research is needed to understand the heterogeneity in patient behavioral and biological responses to exercise in order to have individualized exercise or activity prescriptions that are tailored to the patient/survivor that take into consideration their physical abilities, tolerance to exercise, their cancer stage, status and treatment, their comorbidities, and their likelihood of responding to an exercise regimen. Whilst this objective would be the ideal endpoint, it is unlikely that such level of specificity in the evidence will be achieved in the short term. However, efforts to begin disseminating physical activity guidelines remains paramount given the clear benefits that have already been documented for physical activity after a cancer diagnosis.

In such settings, health professionals play a pivotal role in recommending physical activity to cancer patients and survivors [185]. Nevertheless, safety concerns, time constraints, and the lack of screening tools and referral resources remain barriers for exercise counseling and referral in cancer care [186]. Educational and infrastructure support with brief risk assessment and stratification tools of exercise readiness are needed to accelerate the implementation of existing guidelines. Future studies in these settings should incorporate implementation science methods to optimize the effectiveness and sustainability of the exercise program within complex treatment pathways and health system. Beyond a focus on patient-centered data, implementation process measures with healthcare providers should also be carefully considered using a mixed-methods evaluation approach to understand fully the implementation of exercise programs in the real-world condition. In addition, while exercise programs with professionals specialized in exercise in cancer care exist, this type of resource is often limited. Home-based exercise interventions and other digital exercise options with low resource demands should be developed to meet the needs of the majority of the cancer survivor population [187].

### Natural experiment

While knowledge gaps remain in each of the cancer control categories, there is a strong and growing interest to develop and implement exercise programs in clinical practise [185, 188, 189]. The development of this type of intervention delivery infrastructure can also lead to the creation of natural experiments to generate further evidence on changes in cancer outcomes in response to exposure variations [190]. The traditional approach of translation of evidence into practice takes a linear path that typically starts from proof of concept and feasibility/pilot research, through efficacy and effectiveness studies, to dissemination and implementation trials to generate “evidence-based medicine” [191, 192]. Natural experiments, on the other hand, create the opportunity to evaluate an exercise program rigorously using existing environments, settings, infrastructure to generate “practice-based evidence” that includes all aspects of the research continuum from feasibility to implementation [191]. Such infrastructure will continue to refine the intervention content and offer efficient strategies to gather data on survivors of different types of cancer (e.g., rare cancers) and at various time points in the continuum (e.g., at treatment preparation, treatment effectiveness) that are logistically challenging to study.

## Conclusion

Substantial scientific progress has been achieved in physical activity and cancer control since the publication of the updated PACC framework in 2007. The growing quantity and quality of research has informed the development and refinement of guidelines on physical activity and cancer control for prevention and survival, as well as before, during and after cancer treatment. Nonetheless, significant knowledge gaps remain, particularly in discerning the minimal and optimal dose of physical activity required for each cancer control category and in devising population-based interventions with evidence-based risk stratification strategies tailored to cancer-specific outcomes. This level of evidence is crucial to harness the potential benefit of physical activity fully in reducing the cancer burden. We provided future research directions that incorporate comprehensive monitoring of adverse events, innovative study design, and the application of implementation science to accelerate the translation from evidence generation into practical, real-world interventions.

## Supplementary information

Supplemental Material clean version
