# Supplementary material for: The Physical Activity and Cancer Control (PACC) framework: update on the evidence, guidelines, and future research priorities
Source: Br J Cancer. 2024 Jun 27;131(6):957–69. doi: 10.1038/s41416-024-02748-x (PMC11405831; doi:10.1038/s41416-024-02748-x)
Supplement: Supplementary file 1 — Supplemental Material clean version [file 41416_2024_2748_MOESM1_ESM.docx]

**SUPPLEMENTARY MATERIALS**

We conducted a narrative review to assess the updated current evidence of the role of physical activity in cancer control in accordance with the PACC framework. We searched for published systematic reviews in physical activity/exercise and cancer control in the past five years in MEDLINE by March 19, 2023, and updated the search on April 27, 2024. No search limits were set for study population. The search syntax was limited to cancer (cancer* or malignan* or tumour* or tumor* or metastat* or carcinoma or neoplasm*), physical activity/exercise (exercis* or physical activit* or taiji or tai ji or taichi or tai chi or qigong or qi gong or yoga) and review (review or systematic review or guideline*) combined using an “AND” term. We conducted the literature search using generic terms of physical activity and exercise, as well as specific terms for popular modalities that is outside the scope of conventional exercise, among which yoga, qigong and tai chi are popular options for cancer survivors. We considered any additional reviews based on our knowledge of the literature.

One experienced reviewer (LY) screened all titles and abstracts to remove obviously irrelevant articles and assess for inclusion. Included articles were classified into one or more of the following PACC cancer control categories: primary prevention (reducing the risk of developing a primary cancer), detection (improving the uptake of a cancer screening, sensitivity, or specificity of cancer screening tests), treatment preparation/coping (enhancing health and cancer outcomes prior to receiving cancer treatment), treatment effectiveness/coping (enhancing health and cancer outcomes during cancer treatment), recovery/rehabilitation (improving recovery and rehabilitation from acute impairments and regaining health after cancer treatment), disease prevention/health promotion (enhancing long-term health after rehabilitation including reducing the risk of chronic diseases and cancer recurrence), palliation (improving health among patients diagnosed with advanced cancers), and survival (reducing the risk of death after cancer).

After removing duplicates, a total of 822 articles were retrieved from the initial search, among them 581 were included and 241 articles were deemed irrelevant. Among irrelevant articles, ten did not have English full text, 16 were review protocols, 28 did not report evidence synthesis, two did not review physical activity or exercise interventions, 49 did not review cancer population or cancer relevant outcomes, and 138 reported physical activity or exercise intervention combined with other components. When evaluating the included literature in accordance with PACC cancer control categories, the number of reviews varied significantly, such that 20 articles were focused on primary prevention, one article on detection, 31 on treatment preparation/coping, 73 on treatment effectiveness/coping, 299 on recovery/rehabilitation or disease prevention/health promotion, 22 on palliation, and 15 on survival. In addition, we identified 120 review articles that were a mix of cancer control categories (i.e., reviews of the evidence for the role of physical activity across the cancer control continuum). These numbers of articles derived from one single database (MEDLINE) should not be viewed as a systematic search of all existing literature but can serve as a proxy to reflect the body of research distributed in each PACC cancer control categories.

When systematic reviews were not available for a component of the PACC framework, we included the most current literature based on the best of our knowledge to identify and assess the emerging evidence and to identify future research directions. We included 12 papers that were specifically in the topics of cancer detection. No review protocol was prospectively registered for this narrative review.
